# The Legacy of Shanti Teresa Lakra and Its Impact on Public Health in the Andaman Islands

**DOI:** 10.7759/cureus.67159

**Published:** 2024-08-18

**Authors:** Mitul Saha, Sonali G Choudhari, Swarupa Chakole, Sana Ahmed

**Affiliations:** 1 Department of Community Medicine, Jawaharlal Nehru Medical College, Datta Meghe Institute of Higher Education and Research, Wardha, IND

**Keywords:** padma shri, florence nightingale award, jarawa tribe, tsunami, particularly vulnerable tribal groups, ongee tribe, historical vignette

## Abstract

In the Andaman and Nicobar Islands, Shanti Teresa Lakra, a committed Indian medical nurse, has made a major impact on public health. Lakra, who was born in Rangat on May 1, 1972, was motivated to become a nurse by her elder sister. Her work with the Onge tribe has earned her recognition, particularly in the wake of the 2004 tsunami that destroyed their settlements. Lakra has devoted her professional life to enhancing the health of these indigenous people and averting their extinction by working with particularly vulnerable tribal groups. When she started her work, there were just 78 Onge people living there. She worked constantly to improve healthcare and education, and in five years, the population grew to 100. Her effort required overcoming socioeconomic obstacles, linguistic limitations, and the tribe's initial apprehensions. Despite hazardous circumstances, Lakra helped by immunizing the Jarawa tribe during the COVID-19 outbreak. Her efforts have been recognized with prestigious awards, including the Florence Nightingale Award and the Padma Shri. Her legacy is marked by her empowerment of tribal communities, her role as a healthcare role model, and her advancements in public health in remote areas.

## Introduction and background

**Figure 1:**
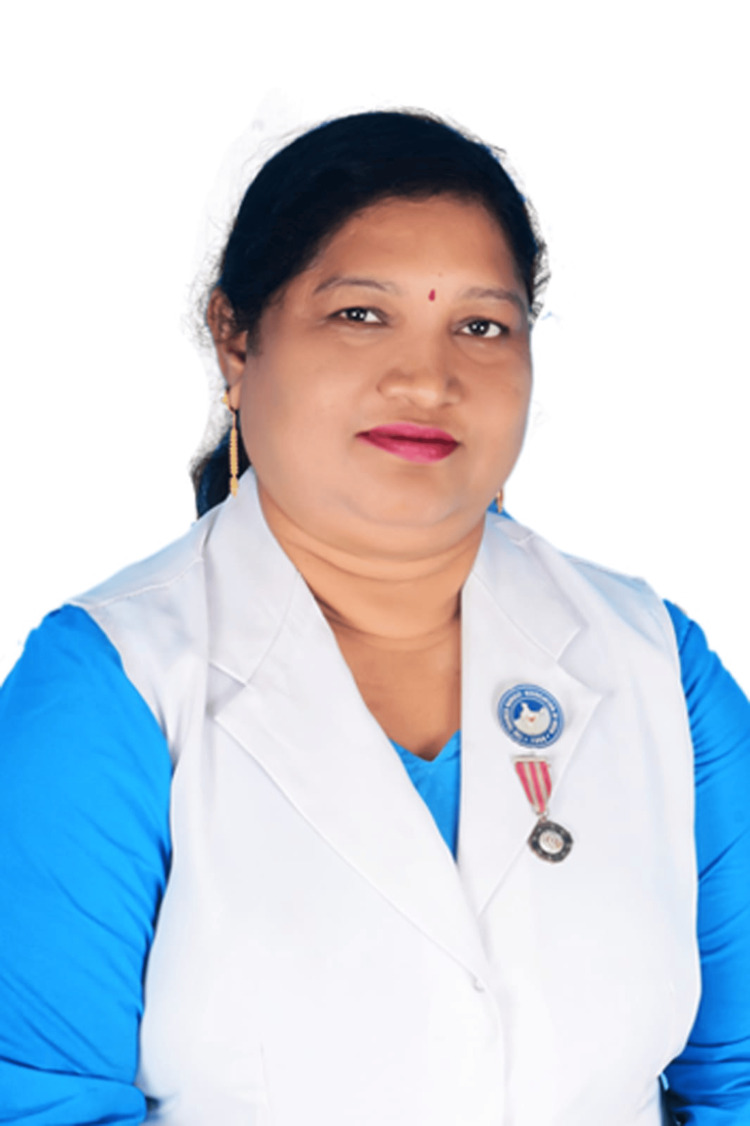
Shanti Teresa Lakra Permission to publish the photograph has been obtained from Shanti Teresa Lakra

Indian nurse and healthcare worker Shanti Teresa Lakra (Figure 1) was born on May 1, 1972, in the tiny village of Rangat in the Middle Andaman, Andaman, and Nicobar Islands [1]. Her mother was from Jharkhand, and her father was from the Sundargarh district of Orissa. Inspired by her older sister, Lakra decided to become a nurse [2]. She gained most of her renown for helping Andaman’s Onge tribe following the 2004 tsunami [3]. Working with particularly vulnerable tribal groups in India’s Andaman and Nicobar Islands, Lakra is devoted to helping the locals and ensuring that poor health conditions don't cause them to go extinct [4]. Lakra has constantly gone above and beyond the call of duty, frequently navigating hazardous situations to make sure that these remote Onge communities receive the care they require. Her work is a testament to the effectiveness of community-based healthcare.

## Review

Tribal health in India and its challenges

The tribal population is 8.6% in India. Tribal healthcare has largely been confined to rural healthcare settings despite long-standing suspicions that these people have significant unmet needs leading to poor health. The unmet healthcare needs in tribal communities can be attributed to various factors such as their social systems, distinct lifestyles, and topography. It is unsurprising that these disparities have not been addressed [5]. The tribal healthcare system in India encounters distinct challenges that differ from those faced by the nation's nontribal healthcare systems. Despite admirable efforts, a number of challenges stand in the way of successfully providing healthcare services to these marginalized groups. These challenges include the need for cultural sensitivity and the incorporation of traditional therapeutic methods, cultural and geographical isolation, barriers arising from language and culture, socioeconomic disparities, and a scarcity of medical personnel. The government, medical professionals, and the native tribes themselves must work together to overcome these obstacles. Their settlements are frequently found in isolated, difficult-to-reach places, such as steep terrain, deep forests, or areas with poor transportation infrastructure. These regions' distinct topography makes it challenging to build and maintain healthcare facilities and guarantee the prompt and effective supply of medical supplies and services [6].

The primitive tribes of Andaman

Enchanting and rich in biodiversity, the Andaman and Nicobar Islands, located in Southeast Asia near India's Bay of Bengal, are a treasure trove of the 2,000-year-old history of human evolution. These islands are home to six distinct tribal groups; five of which are classified as primitive tribes. These primitive tribes are divided into two groups: the Great Andamanese, Onge, and Shompens, who are friendly and in contact with civilization. The second group, the Shompens and Jarawa, is referred to as hostile [7].

Onge tribes of Andaman Nicobar

The Onge (sometimes spelt Ongee, Öñge, and Önge) are an indigenous group native to the Andaman Islands. They are recognized as an Indian Scheduled Tribe [8]. They belong to the "Negrito" ethnic stock with curly black hair, dark skin, and red eyes. They spoke a primitive language that Lakra did not comprehend at the time [3]. Shanti Lakra has spent the last 22 years working with and raising awareness among the primitive tribes who live in remote areas [2]. When she began working with the Onge tribe, there were just 78 members. The men from the tribe often had respiratory tract infections brought on by chewing tobacco. Also, a great number of newborns and women lost their lives giving birth. Fortunately for the tribe, the Ongee population increased to 100 within 4-5 years [3]. The remaining individuals are currently confined to two reserve camps in Little Andaman, South Bay, and the Dugong Creek in the northeast [8]. Lakra mentioned how socioeconomic and linguistic hurdles make her job on the island challenging but rewarding at the same time. “They are very shy by nature, and it isn’t easy for them to share information about their health problems,” she said [7].

Early life and career

Shanti Lakra began working as an auxiliary nurse and midwife at the Directorate of Health Services, Andaman, and Nicobar Administration, in 2001 after completing her nursing studies. She was first posted at the Dugong Creek’s public health center, the land of the Onge tribe. She spent five years working there, during which the 2004 tsunami devastated the settlements. Working with the Onge people, Lakra, a health worker with UNICEF training, is known to have improved the life expectancy of the declining Onge population [1]. When the tribal people visit medical facilities, there is a language barrier, and they are ill-informed about medical procedures. Additionally, their medical histories are unknown. To ensure their comfort, Lakra stayed in their village for a while. Lakra was transferred to Port Blair’s G.B. Pant Hospital towards the end of 2006. She worked in the Special Ward, which is explicitly designated for partially vulnerable tribal groups who are referred from various primary health centers in remote and tribal areas. Together with other staff from the Andaman Adim Janjati Vikas Samiti, she provided constant watch and ward duty in the tribal ward. She took care of patients by managing their hygiene, providing food and clothing, making beds, and escorting them to referred doctors and for various tests such as X-rays, ECGs, ultrasounds, CT scans, and MRIs [9].

Lakra’s contribution to maternal and child health of the Onges

Shanti Lakra lived with the isolated Onge tribe for five years. She cooked and fished with them and witnessed how differently they raised their offspring. One of the reasons for the Onge population being only 78 at the time was the low birth rate. Thus, Onge resolved to prioritize having healthy pregnancies. The Onge do not follow the modern calendar system, making it difficult for women to track their menstrual cycles or confirm pregnancies. They believed acknowledging a pregnancy before it became apparent could bring bad luck [9]. The timid tribal people never talked about their concerns, especially those pertaining to pregnancy, so the nurse had to visit every home. Lakra said that the pregnant women never allowed them to touch their stomachs during a checkup, and she had to persuade them a lot to provide medical care for them [10].

Lakra addressed these issues by simply spending hours in each home, discussing the right and wrong practices of early pregnancy with women. They started seeking her help for basic ailments, first aid, and other medical needs. The tribal people were welcoming and gradually trusted her [11]. An Onge mother who was expecting gave birth to a baby that weighed only 900 grams amid all the devastation caused by the tsunami. She had to save both the mother and the baby, keep them warm, and practice kangaroo mother care for the baby. With limited firewood, Lakra and her team managed. After they had established communication, she asked for a chartered flight from Govind Ballabh Pant Hospital, the closest reference hospital in Port Blair. She had minimal hope for their survival, but six months later, they came back healthy. She conducted three to four deliveries in a single night, all by herself [9]. A total of 102 Ongees are currently living on the island, compared to just 78 when Lakra initially visited [10].

Lakra’s contributions during the tsunami of 2004

**Figure 2:**
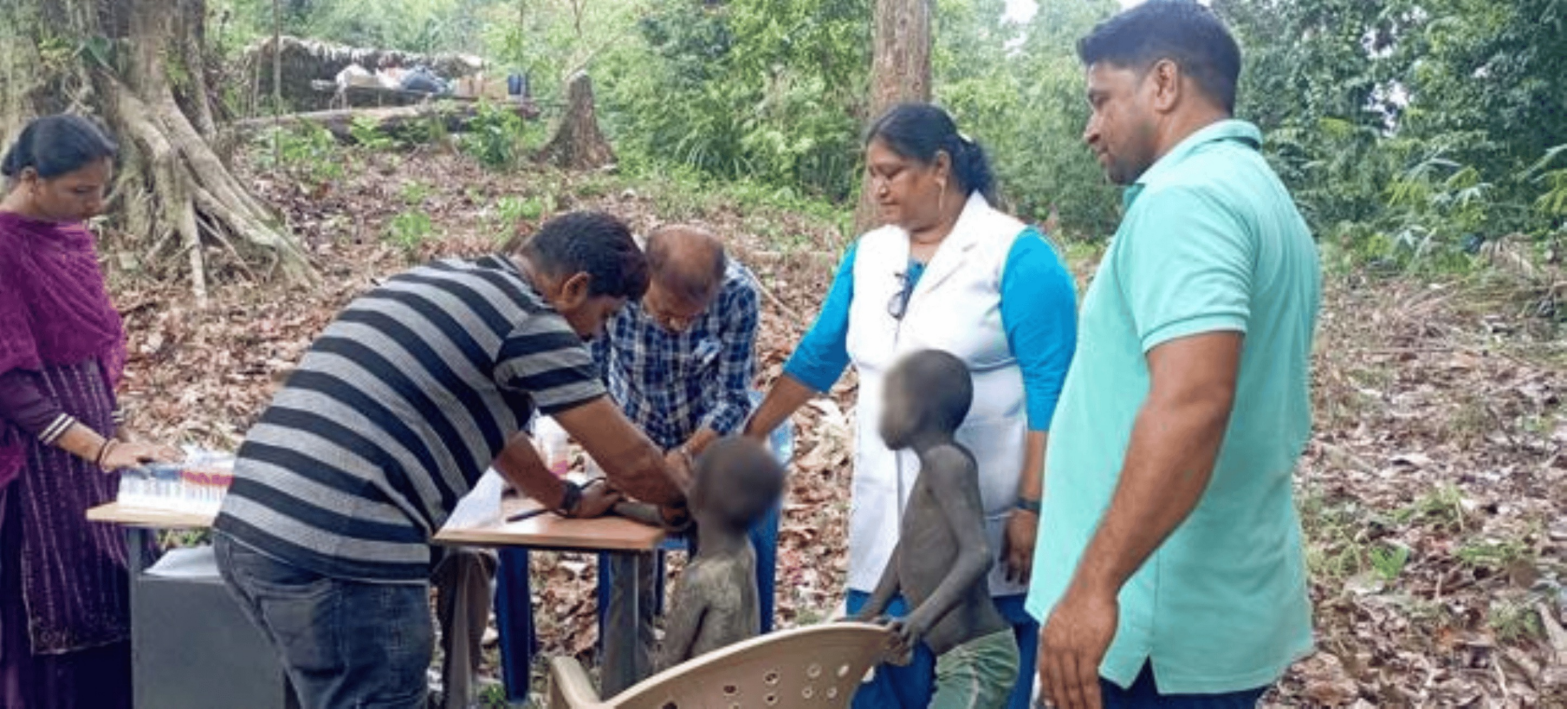
Shanti Teresa Lakra and her team providing healthcare to young Onge tribes Credit: Permission to publish this photogragh has been obtained from Shanti Lakra

The Ongees were driven far into the forest when the 2004 tsunami struck the Andaman coast and habitat of Ongee. Lakra settled in with them and inhabited an open tent [7]. At the time, there were less than 100 Onge, so Lakra committed her life to their health, even though, at first, they were apprehensive about receiving outside assistance. Despite their lack of access to medical records and language challenges, the auxiliary nurse made frequent visits, got to know them, and persuaded them to use the center's medical services. She describes the horrific devastation and difficulties she encountered following the tsunami. The tsunami had washed out the entire Dugong Creek’s settlement, and the Onge were forced to live in a temporary tent and flee farther into the forest [9]. Lakra was determined and settled in with them, living in an open tent [5]. For a considerable amount of time, there was no access to medical supplies or outside communication. She had to search far and wide for medications when the tsunami brought with it a host of illnesses and stress. She sent messengers to walk almost 15 km to bring emergency medicines for the Onge [9]. She continued to live with the Onges for an additional two years, during which time she was unable to visit her family. As the Onges moved deeper into the forests, they frequently undertook long walks, waded through the sea during high tide, and navigated dense forests to deliver healthcare and rehabilitation (Figure 2). They were determined not to abandon the Onges without assistance [9].

Role in COVID-19 vaccination

There is a long history of disease-related deaths among the remote indigenous communities of the Andaman and Nicobar Islands. According to historical accounts, infectious diseases including influenza, measles, mumps, and pneumonia were brought in by outsiders and were a key factor in the 1800s extinction of over half of the native population. Ten members of the Great Andamanese community tested positive with COVID-19 in August 2020. Twenty percent of the Andaman Islands' primitive tribes have COVID-19 infection in 2021. The indigenous people of India are mostly located in rural areas with little access to healthcare. They also don't have timely awareness to cope with the COVID-19 epidemic effectively. Due to their limited contact with the outside world, members of indigenous cultures have not been exposed to novel external infections. They are therefore more susceptible to a range of viral illnesses. Furthermore, the indigenous communities are particularly susceptible to the pandemic due to their limited access to clean water, sanitary conditions, personal protective equipment, and proper healthcare facilities [12]. During the COVID outbreak, Lakra braved cyclonic tides and took a five to six-hour boat ride to reach Tirur Island, where she administered vaccinations to the primitive Jarawa tribe in an effort to save them from extinction in 2021 [9]. Once, Shanti, who was in charge of a small medical team, was afraid that they wouldn't make it when their dinghy got caught in a storm in the Andaman Sea when they were sailing through severe waters. “The sea was extraordinarily rough, and we thought we were all going to drown … But God willed it otherwise, and we managed to reach and vaccinate the Jarawas successfully. We also managed to explain to them the precautionary measures they have to take during the pandemic,” Lakra told the Press Trust of India (PTI) from London [13].

The Jarawas, who have little interaction with outsiders, were thought to be especially susceptible to the illness since, even in the best of circumstances, the tribe has little access to medical care. Lakra has given the Andaman and Nicobar Islands' indigenous tribes devoted service over the years [14]. With the efforts of Lakra and her team, the Onge had managed to isolate themselves well and were taking good care. However, she and her team’s main goal was to get them vaccinated quickly so that the disease would not spread further. She understood that it was important to respect the Onge tribe's culture, beliefs, and lifestyle. She feels relieved to find that the majority of tribes are now accepting and seeking out medical care. Women routinely go to prenatal checkups. There’s also a difference in the weight of babies born, from less than 2 kg to around 2.5 to 2.75 kg, all very good signs [9].

Awards and recognition

**Figure 3:**
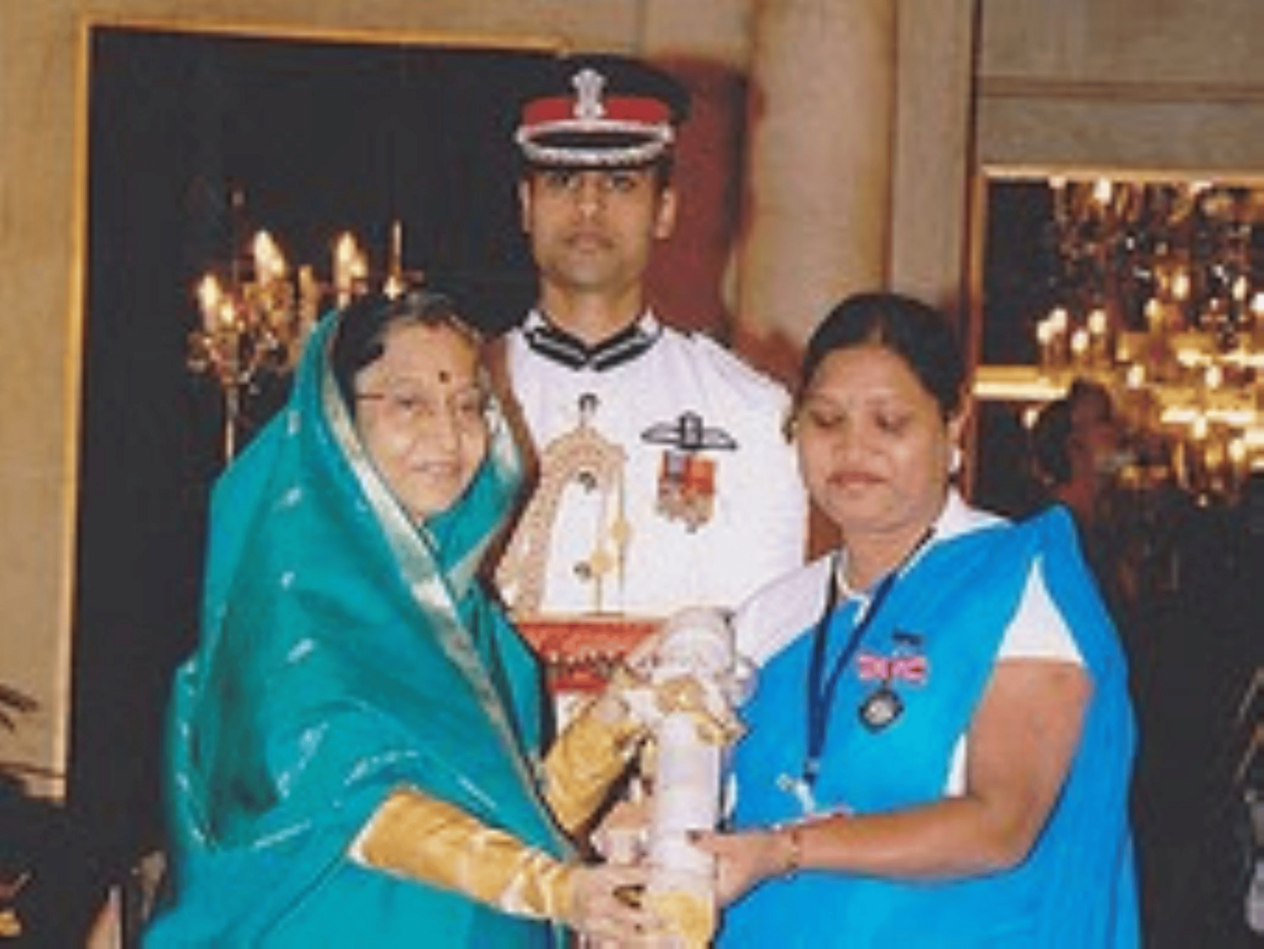
Shanti Teresa Lakra receiving the Padma Shri Award from Honorable President of India, in 2011 Credit: Permission to publish this photograph has been obtained from Shanti Lakra

In 2010, she received the Florence Nightingale Award, a prestigious award for nurses, for her work with the Onge people, which was remarkable, improved their life expectancy, and brought hope to their community [13,15]. She was conferred with the Padma Shri from the then President Pratibha Patil in 2011 (Figure 3) in recognition of her years of nursing work and the significant amount of time she spent in developing trust with the indigenous tribe and helping with their medical requirements [10]. She was among the top 10 finalists for the 2023 Aster Guardians Global Nursing Award [13], for which she was chosen from 52,000 entries sent by 202 countries [14].

Legacy and impact

*Empowerment of Tribal Communities* 

Lakra's work has empowered tribal communities by improving their access to healthcare and raising awareness about health issues. Her efforts have helped build trust between the healthcare system and these communities.

Role model for healthcare workers and advancement of public health in remote areas: Through her service and dedication, she has set a high standard for healthcare professionals, inspiring many to work in underserved areas. She exemplifies compassion and commitment to the welfare of others. Through her relentless efforts, Lakra has made significant strides in public health in the remote regions of the Andaman and Nicobar Islands. Her legacy continues to drive advancements in healthcare delivery in equally challenging environments.

## Conclusions

Shanti Teresa Lakra's exceptional efforts have uplifted the Onge people's standard of living and provided them with hope for the future. Despite the language barrier, she persisted in her efforts to improve the lives of marginalized tribal communities and earned the respect of the reserved Indigenous people. Her dedication to nursing has greatly improved many lives and highlights compassion's essential role in healthcare. Over the years, she gained their trust by providing medical assistance whenever needed and overcoming the language barrier.
